# Evaluation of Changes in Preoperative Cortical Excitability by Navigated Transcranial Magnetic Stimulation in Patients With Brain Tumor

**DOI:** 10.3389/fneur.2020.582262

**Published:** 2021-01-22

**Authors:** Iuri Santana Neville, Alexandra Gomes dos Santos, Cesar Cimonari Almeida, Cintya Yukie Hayashi, Davi Jorge Fontoura Solla, Ricardo Galhardoni, Daniel Ciampi de Andrade, Andre Russowsky Brunoni, Manoel Jacobsen Teixeira, Wellingson Silva Paiva

**Affiliations:** ^1^Instituto do Cancer do Estado de São Paulo, Hospital das Clínicas da Faculdade de Medicina da Universidade de São Paulo, São Paulo, Brazil; ^2^LIM-62/Division of Neurosurgery, Department of Neurology, Faculdade de Medicina da Universidade de São Paulo, São Paulo, Brazil; ^3^Service of Interdisciplinary Neuromodulation, Instituto de Psiquiatria do Hospital das Clínicas da Faculdade de Medicina da Universidade de São Paulo, São Paulo, Brazil; ^4^School of Medicine - Universidade da Cidade de São Paulo UNICID, São Paulo, Brazil

**Keywords:** motor outcome, glioblastoma, neuromodulation, brain tumor, transcranial magnetic stimulation, cortical excitability

## Abstract

**Background:** This prospective study aimed to evaluate the cortical excitability (CE) of patients with brain tumors surrounding or directly involving the **corticospinal tract** (CST) using navigated transcranial magnetic stimulation (nTMS).

**Methods:** We recruited 40 patients with a single brain tumor surrounding or directly involving the CST as well as 82 age- and sex-matched healthy controls. The patients underwent standard nTMS and CE evaluations. Single and paired pulses were applied to the primary motor area (M1) of both affected and unaffected cerebral hemispheres 1 week before surgery. The CE parameters included resting motor threshold (RMT), motor evoked potential (MEP) ratio for 140 and 120% stimulus (MEP 140/120 ratio), short-interval intracortical inhibition (SICI), and intracortical facilitation (ICF). Motor outcome was evaluated on hospital discharge and on 30-day and 90-day postoperative follow-up.

**Results:** In the affected hemispheres of patients, SICI and ICF were significantly higher than in the unaffected hemispheres (*p*=0.002 and ***p***=0.009, respectively). The 140/120 MEP ratio of patients' unaffected hemispheres was lower than that in controls (*p*=0.001). Patients with glioblastomas (GBM) had a higher interhemispheric RMT ratio than patients with grade II and III gliomas (***p*** = 0.018). A weak correlation was observed among the RMT ratio and the preoperative motor score (*R*^2^ = 0.118, *p* = 0.017) and the 90-day follow-up (***R***^**2**^ = 0.227, *p* = 0.016).

**Conclusion:** Using preoperative nTMS, we found that brain hemispheres affected by tumors had abnormal CE and that patients with GBM had a distinct pattern of CE. These findings suggest that tumor biological behavior might play a role in CE changes.

## Introduction

Developed in 1985 by Barker and colleagues (1), transcranial magnetic stimulation (TMS) is a non-invasive, economical, accurate, and well-tolerated method of adjuvant intervention utilized in various neuropsychiatric disorders including major depression (2), Alzheimer's disease (3), diffuse axonal injury (4, 5), schizophrenia (6), and anxiety (7). In neuro-oncology, navigated TMS (nTMS) has been useful in studying electrophysiology in patients with tumors located in eloquent areas to assess motor tract integrity. Brain mapping with nTMS has been associated with a decreased risk of new postoperative neurological deficits and an increased extent of resection (EOR) (8), which are essential for achieving better progression-free survival and quality of life (9, 10).

It has been suggested that preoperative nTMS results could be used as a predictor of motor outcome in patients with lesions involving the primary motor cortex (M1) and corticospinal tract (CST). For example, an abnormal interhemispheric resting motor threshold (RMT) ratio was found to be a high-risk criterion for early poor postoperative motor outcome (7 days), but not for late outcome (3 months) (11). Recent reports also indicate the correlation of the absence of intraoperative motor evoked potential (MEP), detected by postoperative nTMS, with poor motor prognosis (12). In addition, several parameters of cortical excitability (CE), such as short-interval intracortical inhibition (SICI) and intracortical facilitation (ICF), have been described in patients with traumatic brain injury (4) and stroke (13); however, they have not been evaluated in patients with brain tumors involving the M1. Further, the association of abnormal values obtained by nTMS with motor dysfunction is not yet clear.

The aim of the current study was to characterize the CE of patients with brain tumors surrounding the rolandic area and to compare it with those of healthy controls. This would aid in the understanding of how neoplasm behavior affects the neurophysiology of the perilesional motor cortex, using preoperative nTMS.

## Methods

### Setting

For this exploratory prospective study, we recruited 40 adult patients (age ≥18 years old), both genders, with a single brain tumor surrounding or directly involving the CST—a convenience sample—and 82 age- and gender-matched healthy controls. All participants underwent nTMS and CE evaluations at a tertiary referral hospital of São Paulo, Brazil.

### Preoperative Clinical Evaluation

Muscle strength and performance scales were assessed preoperatively, at hospital discharge, and 30-day and 90-day postoperatively. Motor score was defined as upper plus lower extremity strengths of each hemibody according to the Medical Research Council (MRC) (14, 15) grade scale, with 0 indicating no muscle activation and five indicating total muscle strength. Performance status was evaluated using the Karnofsky Performance Scale (KPS) (16). Although the use of antiepileptic drugs (AED) and antidepressants had been previously associated with alterations in neuroexcitability, these drugs could not be withdrawn before nTMS sessions. Instead, we studied the interference of these drugs on CE.

### Brain Tumor Management

Brain tumor diagnosis was established based on clinical history, preoperative magnetic resonance imaging (MRI) analysis, and histopathologic study of each lesion, as per the latest World Health Organization Classification of Tumors of the Central Nervous System (17). Surgical resection was aimed at achieving the best possible EOR.

We used an axial T2-weighted fluid attenuated inversion recovery (FLAIR) MRI sequence to assess the distance (mm) between cortical lesions and the posterior border of the “omega,” correspondent to the area of the hand on the pre-central gyrus (M1). For subcortical lesions, we calculated the distance between the lesion and the posterior limb of the internal capsule.

### nTMS Evaluation

Up to 1 week before surgery, neuronavigation was performed using a frameless stereotaxic system, combining preoperative structural MRI and a sensor-based navigation system (Brainsight TMS version 1.7, Canada) for the guidance of coil placement and visualization of the angle of impact for the magnetic impulse onto the cortical surface (18, 19). Both single- and paired-pulse TMS were applied to M1 of affected and unaffected hemispheres using a circular coil connected to an offline electromyography amplifier of a one-channel, three-surface electrode output (Magventure Tonika Elecktronic, Denmark). The MEP response curve amplitudes were recorded in microvolts (μV) for the first interosseous muscle of the contralateral hand. All evaluations were performed by the same examiner.

CE assessed RMT (%), defined as the lowest stimulus provoking a MEP of at least 50 μV in five out of 10 consecutive trials using single-pulse TMS (20). To assess the amplitude of the input/output curve, we used the MEP obtained with 120 and 140% of RMT stimulus, the most varied range of this curve, and calculated the MEP 140/120 ratio (21–24). With the conditioning stimulus set at 80% of RMT and the test stimulus set at 120% of RMT, we applied paired-pulse TMS and measured SICI by taking the ratio between the amplitude of MEP response curves at 2 and 4 ms inter-stimulus intervals (ISI), while the ICF ratio was calculated taking the ratio between the amplitudes of MEP response curves at ISI 10 ms and 15 ms, for each hemisphere (4, 21, 25). All parameters were classified as low, normal, or high, based on normative values obtained by Cueva *et al*. (25). The ratios between affected and unaffected hemispheres for each parameter were calculated, considering the normal reference range as 90–110%.

### Ethical Standard

This study was approved by the Ethics and Research Committee of the University of São Paulo Medical School, and all individuals provided written informed consent, following the Declaration of Helsinki guidelines.

### Statistical Analysis

Continuous variable normality was verified using the asymmetry and kurtosis values. We performed a Wilcoxon test to compare CE between the affected and unaffected hemispheres. Additionally, to compare the patients with controls, we calculated the mean scores for the controls' hemispheres and compared them with scores for both affected and unaffected hemispheres of all patients using the Mann–Whitney *U* test. Comparisons among the subgroups of patients according to brain tumor histopathology diagnosis (primary central nervous system [CNS] tumor vs. metastasis; World Health Organization [WHO] grade II and III gliomas vs. glioblastomas [GBMs]) were performed using the Mann–Whitney test. We studied the association between motor score and neurophysiological parameters using Spearman's correlation coefficients for quantitative data (absolute value) and Pearson's Chi-square test for qualitative data: classification normal × altered (high + low). Finally, we compared pre- and postoperative muscle strength and KPS using ANOVA for repeated measures. The analyses were performed using the Statistical Package for Social Sciences, version 24.0 (IBM Statistics, Armonk, New York, USA). The data were considered significant when *p* was < 0.05.

## Results

Forty patients underwent nTMS analysis. One patient underwent a new nTMS session before undergoing an additional surgery for recurrent GBM resection a year after the first resection, totalizing 41 CE evaluations. The general characteristics of the patients are presented in Table 1. The mean age of the patients was 50.00 ± 15.34 years, 15 females and 25 males. Similarly, the control group had a mean age of 49.72 ± 15.37 (33 females and 50 males). The mean preoperative KPS score was 83.90 ± 13.01 (range, 50–100). The frequent clinical manifestations that were observed included seizures (27 patients, 73.0%) and hemiparesis (10 patients, 31.3%). The median motor score was 10 (8–10). Around 44% of the patients were taking dexamethasone and 80% antiepileptic drugs when submitted to nTMS session. Three patients were using antidepressants, and only one was using a neuroleptic drug at the time of nTMS session. Thirty-one patients presented cortical tumors while nine patients had subcortical lesions. Thirty-five patients had primary CNS tumors, 23 patients had WHO grade II or III gliomas, 11 had GBMs, and six had secondary brain tumors (originating from the lungs, skin, and gastrointestinal tract).

**Table 1:** General sample characterization.

**Variable, *n* (%)**	**Abscense of hemiparesis**	**Presence of hemiparesis**	**Total *n* (%)**	***p***
Age (years)	45.08 ± 15.46	58.53 ± 11.05	50.00 ± 15.34	**0.009**
Male sex	18 (72.0)	7 (28.0)	25 (62.5)	0.154
Left hemisphere affected	13 (72.2)	5 (27.8)	18 (43.9)	0.300
Awake surgery	12 (75.0)	4 (25.0)	16 (43.2)	0.260
Preop use of dexamethasone	10 (55.6)	8 (44.4)	18 (43.9)	0.355
Preop use of antiepileptic drug	21 (65.6)	11 (34.4)	32 (80.0)	0.683
**CLINICAL PRESENTATION**				
Motor score (MRC)	10	8 (5–8)	10 (8–10)	-
Seizure	18 (66.7)	9 (33.3)	27 (73.0)	0.706
Preop KPS	90 ± 6.32	73.33 ± 14.96	83.90 ± 13.01	**<0.001**
**HISTOLOGY**				
Metastasis	3 (50.0)	3 (50.0)	6 (14.6)	0.460
Lung	2 (66.7)	1 (33.3)	3 (50.0)	
Melanoma	1 (50.0)	1 (50.0)	2 (33.3)	
GTI	0	1 (100.0)	1 (16.7)	0.422
Primary CNS Tumor	23 (65.7)	12 (34.3)	35 (85.4)	
**WHO**				
I	1 (100)	0	1 (2.9)	
II	9 (100)	0	9 (25.7)	
III	10 (71.4)	4 (28.6)	14 (40.0)	
IV	3 (27.3)	8 (72.7)	11 (31.4)	**0.002**
Total	26 (63.4)	15 (36.6)	41 (100)	

*MRC, Medical Research Council; CNS, central nervous system; WHO, World Health Organization. Bold values are statistically significant p values (< 0.05)*.

**Figure 1:**
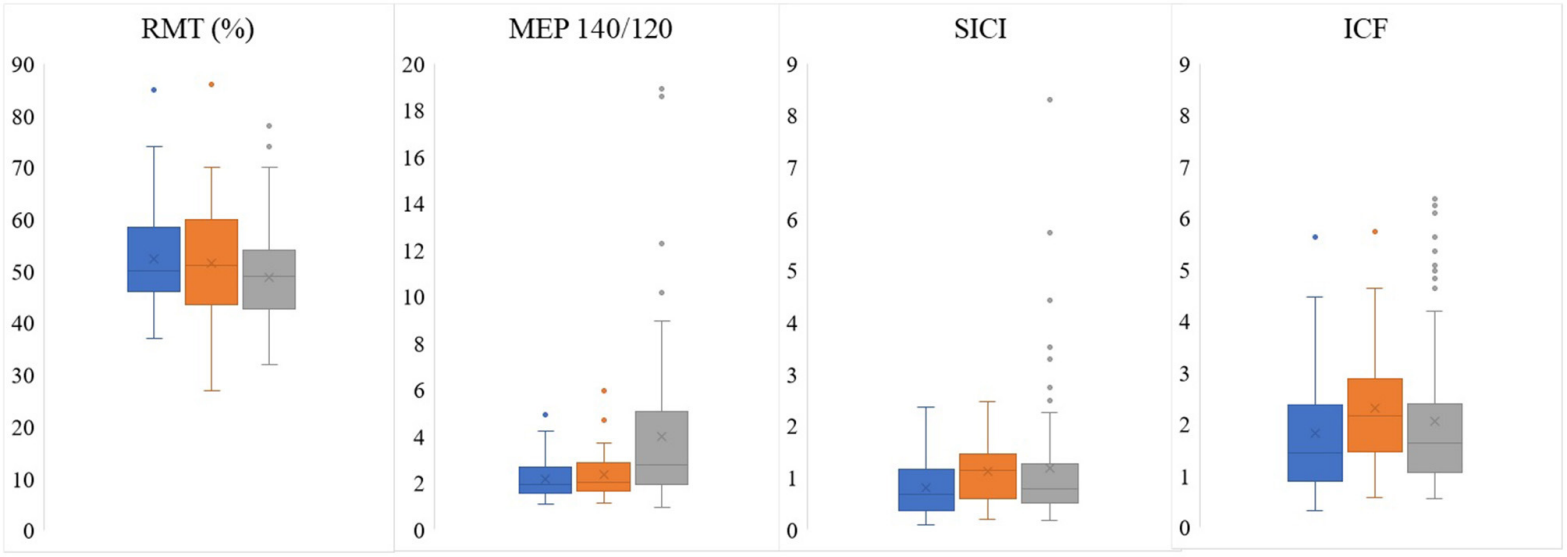
Comparison of electrophysiological parameters obtained by preoperative TMS between unaffected (blue) and affected (orange) patients' hemispheres and the mean between controls' hemispheres (gray). RMT, resting motor threshold; MEP, motor evoked potential; SICI, short-interval intracortical inhibition; ICF, intracortical facilitation.

Assessing CE, we found that SICI and ICF values were significantly higher in the patients' affected hemispheres than in the unaffected hemispheres (1.12 ± 0.60 vs. 0.80 ± 0.59, *p* = 0.002; 2.30 ± 1.14 vs. 1.83 ± 1.20, *p* = 0.009, respectively; Table 2, Figure 1). RMT and MEP interhemispheric ratios exhibited normal distributions in patients, while SICI and ICF interhemispheric ratios had significant interindividual variations. We observed a high frequency of altered (outside the 90–110% range) interhemispheric ratios in the group of patients: 51% of patients had abnormal RMT ratio; 89%, MEP 140/120 ratio; 86%, ICF ratio; and 94%, SICI ratio.

Table 2Transcranial magnetic stimulation parameters in patients and controls.

**Table:** 

**Cortical excitability**	**Patients**		***p* (e)**	**Controls' mean between hemispheres**	***p* (f)**	***p* (g)**
	**Unaffected hemisphere**	**Affected hemisphere**				
RMT(a) %	52.3 ± 10.4	51.4 ± 11.7	0.501	48.7 ± 8.9	0.086	0.176
MEP(b) ratio 140/120	2.15 ± 0.86	2.33 ± 1.04	0.741	3.98 ± 3.41	**0.001**	**0.008**
SICI(c)	0.80 ± 0.59	1.12 ± 0.60	**0.002**	1.18 ± 1.27	0.070	0.191
ICF(d)	1.83 ± 1.20	2.30 ± 1.14	**0.009**	2.05 ± 1.42	0.446	**0.046**

**Table:** 

	**Ratios affected/Unaffected hemisphere**	**Altered (%)**
rRMT	1.0 ± 0.1	19 (51.4)
rMEP ratio 140/120	1.11 ± 0.71	32 (88.9)
rSIICI	1.91 (0.85–3.39)	**35 (94.6)**
rICF	1.29 (0.86–2.27)	32 (86.5)

*RMT(a), resting motor threshold; MEP(b), motor evoked potential; SICI(c), short-interval intracortical inhibition; ICF(d), intracortical facilitation; (e), comparison between healthy and ill hemispheres; (f), comparison between patients' unaffected hemisphere and controls' mean value; (g), comparison between patients' affected hemisphere and controls' mean value. Bold values are statistically significant p values (< 0.05)*.

When the patients were compared to the controls, it was found the MEP 140/120 ratio was lower in patients' both unaffected and affected hemispheres than in those of the controls (3.98 ± 3.41 vs. 2.15 ± 0.86, *p* = 0.001; 3.98 ± 3.41 vs. 2.33 ± 1.04, *p* = 0.008, respectively; Table 2, Figure 1).

The use of antidepressants was not associated with a different CE pattern. Preoperative use of AED seemed not to significantly influence CE in the total population. However, when we studied only the subgroup with CNS tumors, patients who used antiepileptic drugs had significant lower ratio MEP 140/120 and ICF in the affected hemisphere (2.14 vs. 3.54 for MEP ratio, *p* = 0.045, 2.21 vs. 3.42 for ICF, *p* = 0.022, respectively, Supplementary Material 1).

Preoperative clinical and neurophysiologic data of each patient are detailed in Table 3. Thirty-one patients presented abnormal RMT on the unaffected hemisphere and 33 patients presented abnormal RMT on the affected hemisphere. For the other CE parameters, altered MEP 140/120 ratio, SICI, and ICF were more frequent on unaffected hemisphere than the affected one (30 vs. 27 for MEP140/120 ratio, 31 vs. 29 for SICI, and 32 vs. 25 patients for ICF, respectively).

**Table 3:** Preoperative clinical presentation and cortical excitability obtained by navigated transcranial magnetic stimulation of the patients.

**Patient**	**Hemisphere affected**	**UL strength**	**Hemiparesis**	**KPS**	**Histology**	**Distance from motor area**	**Healthy** **hemisphere**				**Affected** **hemisphere**				**Ratio affected/** **healthy hemisphere**			
							**RMT(a) %**	**MEP 140/120 (b)**	**SICI (c)**	**ICF (d)**	**RMT %**	**MEP 140/120**	**SICI**	**ICF**	**RMT %**	**MEP 140/120**	**SICI**	**ICF**
1	Right	4	Yes	70	Anaplastic Astrocytoma	0	37	1.191	0.211	0.382	37	3.283	1.334	3.240	1.0	2.76	6.32	8.48
2	Right	4	Yes	90	Glioblastoma	0	46	2.154	1.792	1.024	55	1.156	0.544	1.094	1.2	0.53	0.30	1.07
3	Right	5	No	90	High Grade Not Otherwise Especified Glioma	0	47	1.607	0.890	2.178	48	2.910	0.921	1.369	1.0	1.81	1.03	0.62
4	Right	5	No	90	Anaplastic Oligodendroglioma	47	69	2.537	0.678	3.043	67	2.604	0.484	2.195	0.9	1.03	0.71	0.72
5	Right	0	Yes	60	Glioblastoma	0	48	1.872	2.356	1.117	64	1.385	1.229	2.651	1.3	0.73	0.52	2.37
6	Right	4	Yes	90	Metastatic Melanoma	0	72	4.230	0.684	2.549	53	4.674	1.590	5.733	0.7	1.10	2.32	2.25
7	Left	5	No	90	Metastatic Melanoma	13,4	38	1.711	0.755	0.890	42	2.013	0.462	1.265	1.1	1.18	0.61	1.42
8	Left	4	Yes	100	Anaplastic Astrocytoma	0	40	2.741	1.651	3.571	43	1.702	1.895	2.163	1.1	0.62	1.15	0.61
9	Left	4	No	80	Glioblastoma	0	48	1.809	0.393	1.937	60	1.571	1.286	1.768	1.2	0.87	3.27	0.91
10	Left	5	No	90	Anaplastic Astrocytoma	33,1	51	1.611	0.531	2.515	55	1.759	0.35	1.482	1.1	1.09	0.66	0.59
11	Left	2	Yes	60	Metastatic GTI Adenocarcinoma	0	74	1.431	0.173	1.189	86	na	1.239	0.798	1.1	na	7.16	0.67
12	Right	5	No	90	Low Grade Not Otherwise Especified Glioma	na	53	1.449	0.880	1.757	49	3.049	0.195	2.258	0.9	2.10	0.22	1.29
13	Right	4	Yes	70	Metastatic Epidermoid Carcinoma (low differentiated)	0	60	1.269	0.367	0.610	61	2.223	0.695	0.577	1.0	1.75	1.89	0.95
14	Left	5	No	90	Metastatic Lung Adenocarcinoma	10,7	40	1.911	0.426	0.597	41	1.359	1.499	1.052	1.0	0.71	3.52	1.76
15	Left	5	No	80	Metastatic Lung Adenocarcinoma	0	46	2.572	0.711	0.864	27	1.787	1.911	2.285	0.6	0.69	2.69	2.64
16	Left	2	Yes	50	Glioblastoma	0	57	1.643	2.186	4.472	na	na	na	na	na	na	na	na
17	Left	5	No	80	Glioblastoma	0	58	1.548	0.819	1.474	63	2.337	1.326	4.330	1.1	1.51	1.62	2.94
18	Left	5	No	90	Anaplastic Astrocytoma	0	63	1.647	0.270	0.545	58	3.611	1.172	4.362	0.9	2.19	4.34	8.00
19	Left	5	No	90	Diffuse Astrocytoma	29,5	57	3.526	1.469	2.233	63	2.335	1.017	2.277	1.1	0.66	0.69	1.02
9	Left	4	Yes	70	Glioblastoma Recurrence	0	53	1.346	0.305	1.225	70	3.474	1.204	2.540	1.3	2.58	3.95	2.07
20	Left	5	No	80	Low Grade Not Otherwise Especified Glioma	0	59	1.159	0.082	1.120	60	1.965	0.790	1.667	1.0	1.70	9.63	1.49
21	Right	5	Yes	70	Anaplastic Oligodendroglioma	0	41	2.356	1.420	3.489	45	1.960	1.050	1.457	1.1	0.83	0.74	0.42
22	Right	5	No	90	Anaplastic Astrocytoma	0	40	2.244	0.337	0.759	46	1.990	0.581	1.746	1.1	0.89	1.72	2.3
23	Right	5	No	90	Diffuse Astrocytoma	0	47	2.793	0.340	1.428	44	1.222	0.692	1.161	0.9	0.44	2.04	0.82
24	Right	5	No	90	Anaplastic Astrocytoma	0	43	3.567	0.672	1.413	33	2.088	0.436	2.155	0.7	0.59	0.65	1.52
25	Left	5	No	90	Anaplastic Astrocytoma	12,1	49	3.157	0.457	2.133	59	3.698	0.574	2.605	1.2	1.17	1.25	1.22
26	Right	5	No	100	Diffuse Astrocytoma	0	61	1.632	0.243	1.379	51	1.225	0.594	1.648	0.8	0.75	2.44	1.20
27	Right	5	No	90	Diffuse Astrocytoma	0	47	1.071	1.281	1.678	36	1.129	1.236	1.938	0.7	1.05	0.96	1.15
28	Left	5	No	90	Anaplastic Astrocytoma	15	50	2.850	0.551	3.754	41	1.757	1.146	2.708	0.8	0.62	2.08	0.72
29	Right	5	No	90	Glioblastoma	20,1	46	2.907	0.445	0.866	45	2.615	2.461	3.359	1.0	0.90	5.53	3.88
30	Right	5	No	100	Diffuse Astrocytoma	15,2	46	2.195	0.154	0.916	46	2.036	0.309	1.475	1.0	0.93	2.01	1.61
31	Right	5	No	80	Meningioma	24,3	50	1.699	0.280	0.322	51	2.573	1.908	4.643	1.0	1.51	6.81	14.42
32	Right	4	No	100	Anaplastic Oligodendroglioma	0	59	2.700	1.370	1.450	na	na	na	na	na	na	na	na
33	Right	5	Yes	90	Diffuse Astrocytoma	56	42	2.145	0.872	3.399	48	5.932	1.402	3.438	1,10	2,77	1,61	1,01
34	Right	2	Yes	80	Glioblastoma	0	65	1.243	1.319	5.631	na	na	na	na	na	na	na	na
35	Right	3	Yes	70	Glioblastoma	9	45	4.896	0.985	1.716	37	2.639	1.992	1.406	0.8	0.53	2.02	0.82
36	Left	5	No	90	Diffuse Oligodendroglioma	0	55	1.229	0.424	1.400	54	1.647	0.811	1.630	1.0	1.34	1.91	1.16
37	Left	0	Yes	50	Glioblastoma	0	85	na	2.135	3.517	na	na	na	na	na	na	na	na
38	Right	4	Yes	90	Glioblastoma	0	51	1.911	1.044	2.159	64	1.254	2.412	3.100	1.2	0.65	2.31	1.43
39	Right	5	No	100	Diffuse Oligodendroglioma	47	54	2.454	0.423	1.438	47	3.458	0.604	2.605	0.8	1.41	1.42	1.81
40	Right	5	No	100	High Grade Not Otherwise Especified Glioma	0	53	2.054	0.567	0.883	54	1.678	2.180	3.076	1.0	0.81	3.84	3.48

*RMT(a), resting motor threshold (%); MEP(b), motor evoked potential; SICI(c), short-interval intracortical inhibition; ICF(d), intracortical facilitation; na, not applicable*.

When we compared patients according to the presence of hemiparesis, the only difference found was a higher SICI in the unaffected hemisphere of patients with hemiparesis (*p* = 0.013). However, there was no difference in interhemispheric SICI ratio (Table 4). Comparing the subgroups of patients according to their histopathological diagnoses revealed no significant difference in CE between patients with primary and secondary tumors. However, comparisons of patients with GBMs with patients with WHO grade II and III gliomas indicated that patients with GBMs had a higher interhemispheric RMT ratio (*p* = 0.018; Table 4). Table 5 shows a detailed analysis of CE in each group of patients, according to their tumor diagnosis.

**Table 4:** Ratio affected/unaffected hemisphere according to the presence of hemiparesis and histology.

**Cortical excitability**	**Presence of hemiparesis**	**Absence of hemiparesis**	***p***	**Primary CNS tumor**	**Metastasis**	***p***
rRMT (a)	1.1 ± 0.2	1.0 ± 0.1	0.075	1.0 ± 0.1	0.9 ± 0.2	0.635
rMEP ratio 140/120 (b)	1.21 ± 0.85	1.18 ± 0.56	0.458	1.20 ± 1.13	1.08 ± 0.43	0.909
rSICI (c)	2.02 (0.74–3.95)	1.81 (0.89–3.33)	0.842	1.72 (0.74–3.27)	2.50 (1.57–4.43)	0.303
rICF (d)	1.07 (0.67–2.25)	1.35 (0.98–2.38)	0.425	1.22 (0.82–2.30)	1.59 (0.88–2.34)	0.805
	**Low-grade gliomas**	**High-grade gliomas**		**WHO grade II-III glioma**	**Glioblastoma**	
rRMT (a)	0.9 ± 0.1	1.0 ± 0.1	0.078	1.0 ± 0.1	1.1 ± 0.2	**0.018**
rMEP ratio 140/120 (b)	1.41 ± 0.68	1.10 ± 0.67	0.367	1.25 ± 0.68	1.04 ± 0.69	0.270
rSICI (c)	1.61 (0.82–2.22)	1.72 (0.72–3.55)	0.860	1.52 (0.73–2.17)	2.16 (0.79–3.78)	0.482
rICF (d)	1.20 (1.08–1.55)	1.22 (0.72–2.65)	0.792	1.18 (0.72–1.66)	1.75 (0.95–2.79)	0.241

*r, ratio affected/unaffected hemisphere; RMT(a), resting motor threshold; MEP(b), motor evoked potential; SICI(c), short-interval intracortical inhibition; ICF(d), intracortical facilitation. Bold values are statistically significant p values (< 0.05)*.

**Table 5:** Comparison between affected and unaffected hemispheres according to the tumor diagnosis.

**Cortical excitability**	**UH**	**AH**	***p***	**UH**	**AH**	***p***
	**Metastasis (*****n*****= 6)**			**Primary CNS tumor (*****n*** **= 35)**		
RMT(a) %	55.0 ± 15.9	51.7 ± 20.4	0.916	51.8 ± 9.4	51.4 ± 9.8	0.363
MEP(b) ratio 140/120	2.18 ± 1.10	2.41 ± 1.30	0.893	2.14 ± 0.83	2.32 ± 1.02	0.799
SICI (c)	0.52 ± 0.23	1.23 ± 0.55	**0.046**	0.85 ± 0.62	1.10 ± 0.62	**0.014**
ICF(d)	1.11 ± 0.73	1.95 ± 1.94	0.173	1.95 ± 1.22	2.37 ± 0.95	**0.031**
	**Low-grade gliomas (*****n*** **= 10)**			**High-grade gliomas (*****n*** **= 25)**		
RMT(a) %	52.7 ± 6.4	50.4 ± 8.0	0.292	51.6 ± 10.6	51.8 ± 10.8	0.108
MEP(b) ratio 140/120	1.87 ± 0.79	2.53 ± 1.49	0.214	2.26 ± 0.85	2.22 ± 0.81	0.476
SICI (c)	0.65 ± 0.50	0.77 ± 0.40	0.374	0.95 ± 0.65	1.20 ± 0.65	**0.039**
ICF(d)	1.70 ± 0.74	2.10 ± 0.62	**0.008**	2.10 ± 1.34	2.38 ± 0.96	0.181
	**WHO grade II and III gliomas (*****n*** **= 23)**			**Glioblastomas (*****n*** **= 11)**		
RMT(a) %	50.6 ± 8.3	49.3 ± 8.8	0.549	54.7 ± 11.8	57.2 ± 11.1	0.050
MEP(b) ratio 140/120	2.17 ± 0.74	2.41 ± 1.11	0.527	2.13 ± 1.07	2.05 ± 0.83	0.401
SICI (c)	0.68 ± 0.46	0.90 ± 0.50	0.060	1.25 ± 0.76	1.55 ± 0.67	0.327
ICF(d)	1.86 ± 1.01	2.21 ± 0.79	0.355	2.28 ± 1.57	2.53 ± 1.08	0.069

*RMT(a), resting motor threshold; MEP(b), motor evoked potential; SICI(c), short-interval intracortical inhibition; ICF(d), intracortical facilitation. Bold values are statistically significant p values (< 0.05)*.

**Figure 2:**
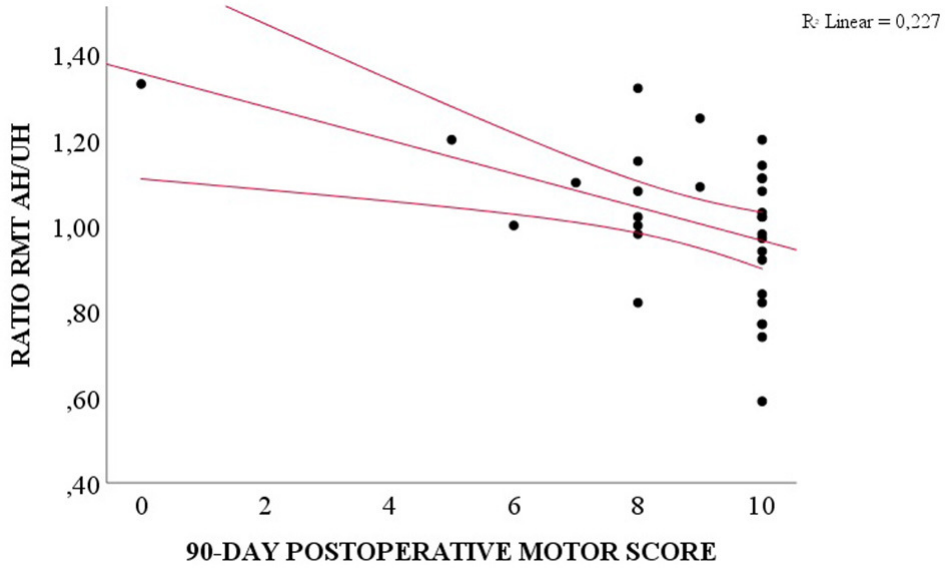
Correlation between preoperative RMT ratio AH/UH and 90-day postoperative motor score (Spearman, *p* = 0.016).

We compared the preoperative and the three postoperative motor evaluations. Patients presented the highest motor score at the preoperative moment and the lowest at the hospital discharge (Table 6, *p* = 0.030). A weak correlation was observed among the RMT ratio and the preoperative motor score (*R*^2^ = 0.118, *p* = 0.017), and the 90-day follow-up (*R*^2^ = 0.227, *p* = 0.016), and between unaffected hemisphere SICI and the pre- and postoperative motor scores (*R*^2^ = 0.255, *p* = 0.009 for preoperative motor score, *R*^2^ = 0.271, *p* = 0.018 for hospital discharge, *R*^2^ = 0.321, *p* = 0.013 for 30-day follow-up, and *R*^2^ = 0.396, *p* = 0.059 for 90-day follow-up, Table 5). However, preoperative RMT ratio and unaffected hemisphere SICI were not associated with motor score change (*p* = 0.938 for RMT and *p* = 0.470 for SICI, ANOVA for repeated measures, Figure 2).

**Table 6:** Correlation of spearman between cortical excitability and motor and performance scale outcomes.

**Variables**	**Mean ± SD**	**Unaffected hemisphere**				**Affected hemisphere**				**Ratio affected/unnaffected hemisphere**			
		**RMT**	**MEP 140/120**	**SICI**	**ICF**	**RMT**	**MEP 140/120**	**SICI**	**ICF**	**RMT**	**MEP 140/120**	**SICI**	**ICF**
**Preoperative**													
MS	10 (8–10)	0.345	0.565	**0.009**	0.161	0.206	0.764	0.099	0.296	**0.017**	0.402	0.919	0.311
KPS	83.90 ± 13.01	0.184	0.060	0.373	0.932	0.162	0.706	0.194	0.530	0.157	0.480	0.187	0.783
**Hospital discharge**													
MS	9 (6–10)	0.683	0.656	**0.018**	0.260	0.961	0.237	0.928	0.078	0.490	0.242	0.176	**0.220**
**30-day follow-up**													
MS	10 (7–10)	0.820	0.136	**0.013**	0.603	0.413	0.339	0.895	0.148	0.055	0.997	0.116	0.655
KPS	78.92 ± 18.67	0.978	0.327	0.109	0.928	0.553	0.986	0.199	0.081	0.213	0.835	0.164	0.744
**90-day follow-up**													
MS	10 (8–10)	0.445	0.398	0.059	0.862	0.516	0.514	0.873	0.156	**0.016**	0.718	0.232	0.717
KPS	82.73 ± 13.29	0.464	0.507	0.247	0.664	0.636	0.812	0.542	0.126	**0.039**	0.364	0.578	0.834

*MS, motor score; KPS, Karnofsky Performance Scale. Bold values are statistically significant p values (< 0.05)*.

A correlation was observed between distance (in millimeters) from motor area on MRI and the MEP 140/120 ratio of both hemispheres (*p* = 0.030, *R*^2^ = 0.348 in the unaffected hemisphere and *p* = 0.032, *R*^2^ = 0.363 in the affected hemisphere).

## Discussion

This study contributes to the knowledge about the neurophysiology of patients with tumors within M1. It evaluates distinct parameters that have been previously reported, RMT and MEP, as well as describes the values of SICI and ICF for the first time. The first two parameters refer to the integrity of both upper and lower motor neurons, from the cerebral cortex to the neuromuscular junction. RMT was similar between patients' hemispheres, which contradicts previous studies that reported higher RMT in the hemisphere affected by the tumor (26). SICI and ICF, the only two parameters found to be significantly higher in patients' affected hemispheres, are exclusively mediated by circuits located in the cortex (27, 28) and, therefore, have a higher specificity for cortical alterations than RMT and MEP. SICI is mediated by GABA_A_ receptors, which are ligand-gated ion channels, while long-interval intracortical inhibition is associated with GABA_B_. These are G-protein-coupled receptors and are, therefore, slower than GABA_A_ (28–30). Varrasi *et al*. found abnormal intracortical inhibition in patients with partial epilepsy, which was attributed to weakness of the GABA receptors, thereby provoking an imbalance between excitatory and inhibitory circuits (31). SICI was also found to be reduced in movement disorders, such as dystonia (32) and Tourette's syndrome (33). Conversely, ICF is mediated by glutamate and is associated with excitatory cortical circuits (34). SICI was found to be lower with a concomitant increase in ICF values in patients with Parkinson's disease (35). In our study, the patients' unaffected hemisphere's excitability was lower compared to that of the controls and compared to the affected hemisphere. Since observations of significant interhemispheric differences in healthy individuals are unexpected (36), the differences found in our study might be associated with dysfunction of motor neurons in the patients' hemispheres affected by the tumor as well as an interhemispheric imbalance between excitatory and inhibitory circuits, which might be related to the greater prevalence of preoperative seizures in our study (73%). The mechanism by which the CE parameters of the contralateral hemisphere is altered as well is still unclear.

It has been reported that the use of antiepileptic, neuroleptic, and antidepressant drugs can affect neuroexcitability (37–41). Voltage-gated sodium channels blockers, such as phenytoin, carbamazepine, and lamotrigine, were previously found to increase motor threshold (41, 42), with carbamazepine associated with decreased ICF (42). In our series, the influence of AED on CE was only observed in the subgroup of patients with CNS tumors, composed of 29 patients who used AED and 6 patients who did not use it. Although the findings of lower ratio MEP 140/120 and lower ICF in the group using AED might agree with previous studies, they also might reflect a type 1 error.

It has been reported that MEP may have high interindividual variability and that the interhemispheric ratio has a more reliable value in assessing CE (26). In our sample, we found high rates of abnormal ratios (51% for RMT, 89% for MEP 140/120 ratio, 86% for ICF, and a remarkable 94% for SICI). However, only half of the patients presented with motor deficits, which led us to two main hypotheses: The first is that the alteration of these values may coexist with a normal motor function because tumor growth is not an acute process, requiring some time to progressively affect the tissue surrounding it. This conclusion applies especially to patients with low-grade gliomas, which have a relatively slower evolution, giving the unaffected brain some time to try to compensate by neuroplasticity. The second hypothesis is that pathologic neurophysiology may predict a poor motor outcome in these patients in the future. Picht et al. speculated that patients initially without hemiparesis but with high RMT and low MEP interhemispheric ratios were at a higher risk of a decline in motor function in the future (26). These authors also suggested that patients with no previous deficits but with high MEP ratios probably have a perilesional tissue more adapted to tumor growth. Additionally, they highlighted the finding that the patients had low RMT ratios, suggesting that in these individuals, tumors might have infiltrated the inhibitory tracts of the secondary motor cortex, thereby accounting for the lack of motor deficits (26). In our study, we found six patients [patients 2, 5, 9, 19, 22, 38] with simultaneously high RMT and low MEP interhemispheric ratios, two of whom had no motor deficits prior to the resection (19, 22). Four patients had worse motor scores during the follow-up (2, 5, 22, 38), one patient remained stable (9), and one of the two patients who initially did not have any motor deficit displayed normal motor function (19). This patient is also the only one in the subgroup with a low-grade glioma, as all the others were diagnosed with high-grade gliomas. These data are consistent with Picht *et al*.'s speculation about poor outcome prediction and our second hypothesis. In our study, 10 patients (3, 7, 12, 17, 18, 20, 25, 31, 36, 39) had high MEP ratios with normal preoperative motor status, and six remained with no motor deficit (7, 18, 20, 25, 31, 39), which again is consistent with the findings of Picht *et al*. and fits our first hypothesis of adaptation of the normal tissue. The direct correlation between greater tumor's distance from motor area and higher MEP also reinforces this hypothesis.

Lastly, we observed eight patients (6, 15, 24, 26–28, 35, 39) with a low RMT ratio, and, contrary to our expectations, six of them had a high SICI ratio (6, 15, 26, 28, 35, 39). Indeed, the only difference between patients concerning hemiparesis presentation was a lower SICI in patients without this motor deficit. Concerning clinical presentation, however, the presence of preoperative motor deficit was not associated with more CE abnormalities.

The idea that preoperative TMS findings might predict motor outcome is not new. Rosenstock *et al*. studied abnormal RMT interhemispheric ratio as one criterion for high risk of poor motor outcome and found that a high RMT ratio was associated with worst motor score 7 days postoperatively, but that there was no association 3 months postoperatively (11). Therefore, more analysis is necessary to determine whether preoperative SICI and ICF are associated with presence of motor deficit at the time of CE evaluation and might predict patients' prognoses.

Tumor growth rate influences the surrounding cortex adaptation, and this could explain another finding of our study, the higher values of RMT ratio in patients with GBMs compared with patients with WHO grade II and III gliomas. It is well-known that GBM rapidly infiltrates parenchyma, hindering motor function recovery. Therefore, GBM affects CE in a way closer to the changes seen in acute/subacute brain injuries. In their meta-analysis, McDonnel *et al*. found that RMT was already higher in affected hemispheres at the early phase after stroke and continued to be altered during the chronic phase (13). As discussed previously, high RMT ratio might be a sign of a decline in motor function, even in those who do not have clinical manifestations.

This is an exploratory study whose findings contribute to the knowledge of how neuroexcitability might be affected by a tumor. However, some results require further studies to be well-understood. One of the limitations of our study is tumor heterogeneity: We included a majority of patients with gliomas (of both low and high grades and, therefore, different rates of normal tissue infiltration), a minority of patients with brain metastases (which typically provoke mass-effect alterations), and one patient with a grade I meningioma, an extra-axial tumor related to alterations due to the tumor's expansion. It is impressive that most CE parameters studied had a normal distribution considering different biological behaviors of different tumors. We had a glance on how CE in each subgroup of diagnosis is. However, focusing our attention on small subgroups increases the risk of a type 1 error. Therefore, these specific data should be considered only for descriptive purposes.

Another potential bias in the study is that the MEPs were only measured on the hands and not also on the lower extremities; more than 63% of patients presented no motor deficit or had a mild deficit preoperatively (motor score of 8–10), reflecting a possible selection bias and hindering the correlation of CE data with motor outcome. The final limitation is the lack of data on cognition and quality of life. Only minor adverse events were observed, such as light pinch on scalp and light headache during nTMS.

This study provides a detailed description of the CE of patients with tumors located in the eloquent areas of the brain. Brain hemispheres affected by tumors had abnormal CE, but further studies are needed to determine if CE is associated with loss of motor function integrity. GBMs showed a discrete pattern when compared with grade II and III gliomas, suggesting that tumor biological behavior might play a role in CE changes observed in patients with gliomas.

## Data Availability Statement

The original contributions generated for this study are included in the article/Supplementary Material, further inquiries can be directed to the corresponding author/s.

## Ethics Statement

The studies involving human participants were reviewed and approved by Comitê de Ética e Pesquisa (Ethics and Research Committee, CEP) of University of São Paulo Medical School. The patients/participants provided their written informed consent to participate in this study.

## Author Contributions

IN, RG, DA, AB, MT, and WP: conception and design. IN: patients recruitment. IN, AG, CA, and CH: data collection. IN, AG, and DS: data analysis and interpretation. IN and AG: drafting the manuscript for important intellectual content. DS, RG, CH, DA, AB, MT, and WP: text review. IN, AG, CA, CH, DS, RG, DA, AB, MT, and WP: final approval. All authors contributed to the article and approved the submitted version.

## Conflict of Interest

The authors declare that the research was conducted in the absence of any commercial or financial relationships that could be construed as a potential conflict of interest.
